# Supplementary material for: One-Pot Synthesis of Cellulose-Based Carbon Aerogel Loaded with TiO2 and g-C3N4 and Its Photocatalytic Degradation of Rhodamine B
Source: Nanomaterials (Basel). 2024 Jul 2;14(13):1141. doi: 10.3390/nano14131141 (PMC11243333; doi:10.3390/nano14131141)
Supplement: Supplementary file 1 [file nanomaterials-14-01141-s001.zip › nanomaterials-3033754-supplementary.pdf]

Supplementary Materials

One-Pot Synthesis of Cellulose-Based Carbon Aerogel Loaded with TiO_2 and $g\text{-}C_3N_4$ and Its Photocatalytic Degradation of Rhodamine B

Fangqin Liu, Mingjie Fan, Xia Liu and Jinyang Chen *

School of Environmental and Chemical Engineering, Shanghai University, 99 Shangda Road, Shanghai 200444, China; fqliu19@shu.edu.cn (F.L.)

* Correspondence: chenjy@shu.edu.cn; Tel.: +86-21-66137729; Fax: +86-21-66137725

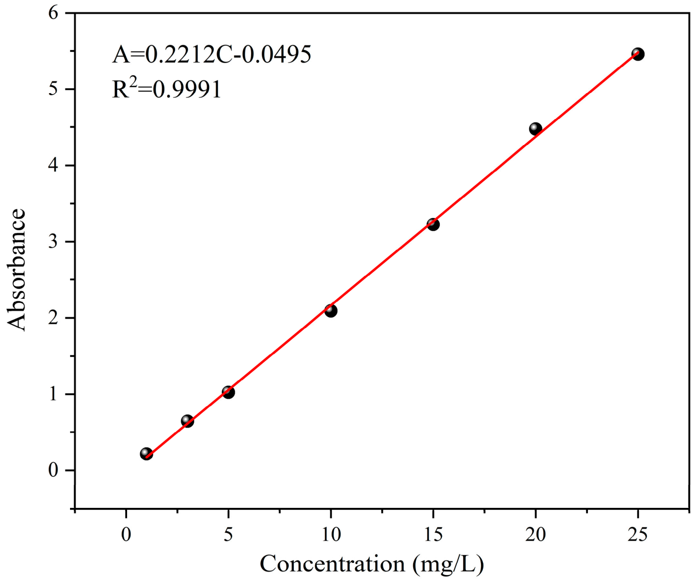

Figure S1. Calibration curve of Rh. B at a wavelength of 554 nm.

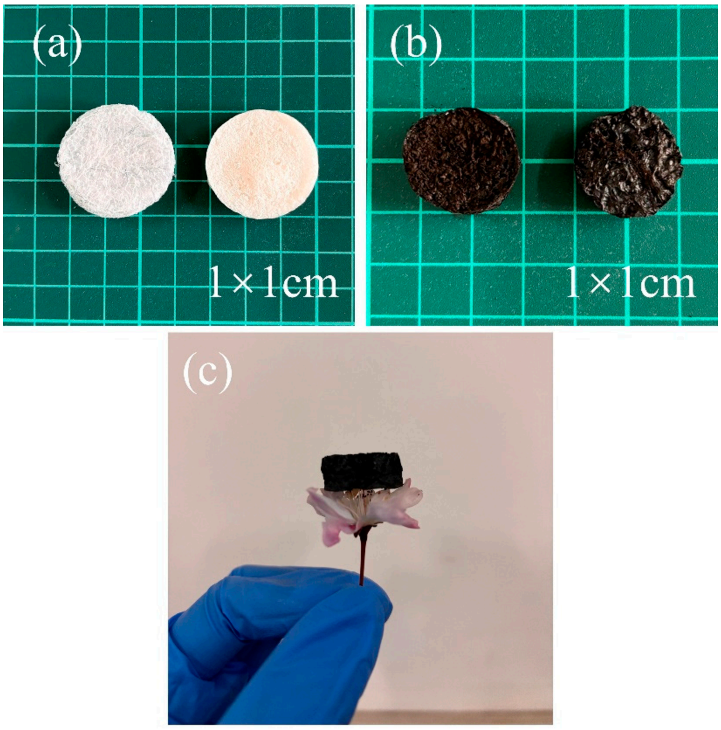

Figure S2. Photos of CTN with ultra-light features (a-c).

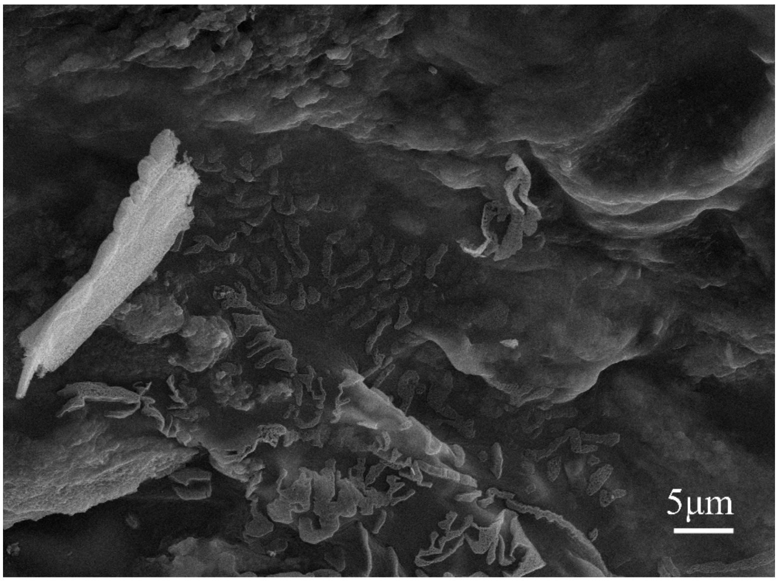

Figure S3. SEM image of CTN.

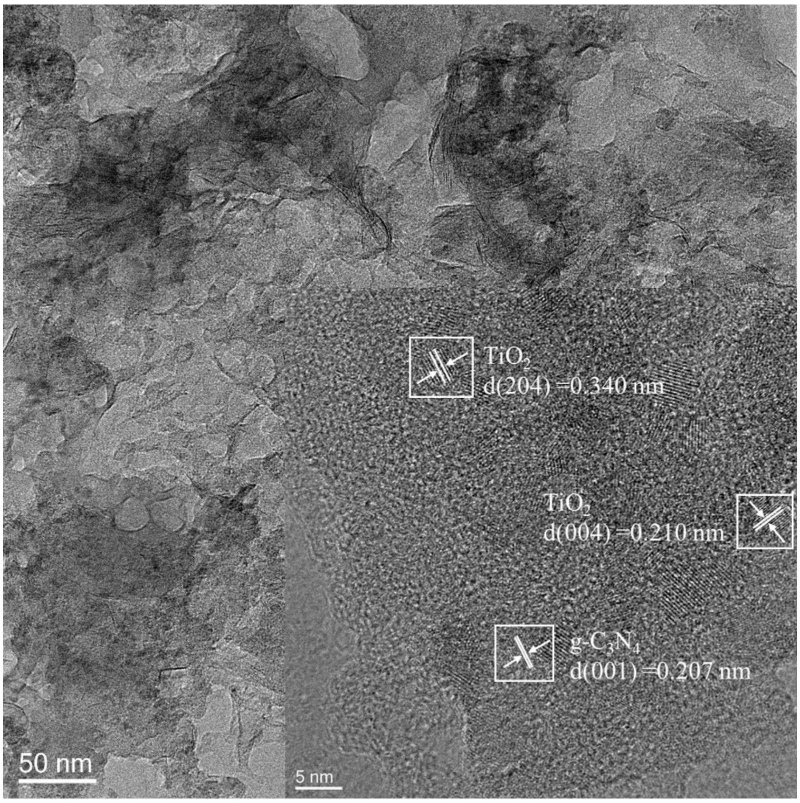

Figure S4. TEM image of CTN.

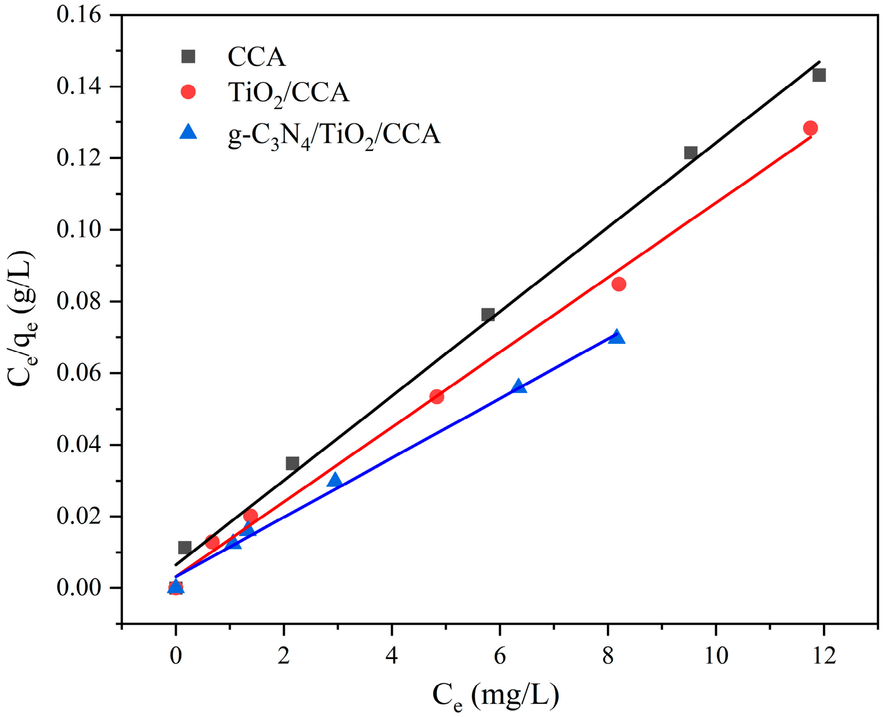

Figure S5. Langmuir adsorption isotherm of Rh. B by carbon aerogels.
